# Supplementary material for: Are carbon emissions trading and green financial instruments synergistic? -Comprehensive quantitative research based on content analysis
Source: PLoS One. 2024 Mar 7;19(3):e0298601. doi: 10.1371/journal.pone.0298601 (PMC10919601; doi:10.1371/journal.pone.0298601)
Supplement: S1 Data — (ZIP) [file pone.0298601.s001.zip › Data/地级市Z_F工作报告.zip!/║■▒▒/╛ú├┼╩╨/└┤╫╘╣1⁄2╓┌║┼í╛┬φ┐╦╩2╛▌═°í┐_2007.docx]

各位代表：

　　现在，我代表市人民政府向大会作工作报告，请予审议，并请市政协各位委员和其他列席人员提出意见。

　　一、市六届人民政府工作的回顾

　　市六届人大一次会议召开四年以来，在省委、省政府和市委的正确领导下，在市人大、市政协的监督与支持下，市政府团结和带领全市人民，高举邓小平理论和“三个代表”重要思想伟大旗帜，全面落实科学发展观，解放思想，开拓创新，克难奋进，在全面建设小康社会的进程中迈出了坚实的步伐。

　　综合经济实力进一步增强。全市生产总值达到348.73亿元，年均增长10.1%。地域财政收入达到25.68亿元，年均增长7.3%。实施“兴工富市”战略，工业经济保持平稳增长，全市规模以上工业增加值年均增长17.6%，规模以上工业企业发展到486家，其中亿元企业53家。中石化荆门分公司年销售收入突破160亿元，京山轻机、湖北洋丰、天茂集团等一批企业不断发展壮大。金龙泉啤酒再次荣获“中国名牌产品”称号。发展后劲逐步增强，累计完成全社会固定资产投资450亿元，年均增长15.2%。荆门电厂三期扩建工程进展顺利，首台60万千瓦机组投入运行；中石化荆门分公司扩能改造项目如期进行；中粮祥瑞、国宝桥米、金龙非织造布、永兴水泥、宝源中高密度纤维板等一批投资过亿元的重点项目相继建成投产。开发区和工业园区建设加快，集聚效应开始显现。农业农村经济稳步发展，新农村建设积极推进。市级以上农业产业化重点龙头企业达到103家，其中国家级、省级重点龙头企业分别为2家、18家。旅游、房地产、金融、保险、交通运输、邮电通信等服务业快速发展，我市被授予“中国优秀旅游城市”称号，连续三年被评为“全省A级金融信用市”。县域经济加快发展，京山、东宝、钟祥2005年进入全省县域经济发展20强。人民生活水平不断提高，全市城镇居民人均可支配收入和农民人均纯收入分别达到9392元、4059元，年均增长9.3%和6.2%，社会消费品零售总额年均增长10.5%。

　　改革开放取得新突破。国企改革向纵深推进，市属企业改制面达98%。荆襄公司改革脱困取得明显成效，凯龙集团、宏图飞机制造厂、东光集团改革进展顺利，在荆中省企业剥离办社会职能基本完成。多方筹资解决企业改制遗留问题。积极探索国资监管体制改革。财政预算管理制度改革和会计管理工作走在全省前列。农村税费改革不断深化，取消农业特产税，免征农业税，土地二轮延包工作顺利完成。乡镇综合配套改革成效显著。完成市、县两级政府机构改革。深化行政审批制度改革，取消和下放审批项目800余项。对外开放水平继续提高，累计完成进出口总额4.6亿美元，实际利用外资3亿美元。

　　城乡面貌明显改观。城市总体规划修编完成。加强城市基础设施建设，完成东宝山隧道、竹皮河城区段治理、夏家湾污水处理厂等一批重点项目建设。中心城区建成区面积达到44.6平方公里，人口40万。钟祥、京山、沙洋县城和一批重点小城镇建设步伐加快，全市城镇化率达到38.6%。襄荆高速公路、长荆铁路、江汉航线投入运营，荆宜、随岳、武荆高速公路荆门段开工建设，二级公路路网建设基本完成，通油路（水泥路）的行政村达62%，全市形成“三纵三横”的交通主骨架。全面完成“户户通电”工程。广播电视实现行政村“村村通”。加强生态建设和环境保护，工业“三废”和生活污水处理率不断提高，漳河水源保护工作有效开展，矿产资源整顿与综合利用扎实推进。综合治理水土流失，全市森林覆盖率达到35.7%。

　　社会事业全面发展。以县为主的农村义务教育管理体制得到落实，“普九”成果进一步巩固提高，高中阶段办学规模不断扩大，职业教育呈现生机。荆门职院与沙洋师专合并筹建本科学院工作进展顺利。科技创新和科技服务体系建设加强，我市连续两次荣获“全国科技进步先进市”称号。建成青少年活动中心、妇女儿童活动中心和青少年科技馆，“八艺节”分会场场馆建设顺利进行，市体育文化中心主体工程完工。文化艺术、新闻出版、广播电视、社会科学等领域的一些优秀作品在全国、全省获奖，《十二月等郎》获全国地方戏优秀剧目展评金奖第一名。群众性体育活动蓬勃开展，竞技体育取得新突破，何军权在雅典残奥会上勇夺4枚金牌。成功抗击“非典”、禽流感疫情，艾滋病、结核病、血吸虫病等重大传染病和地方病的防治得到加强。积极推进新型农村合作医疗试点工作，参合农民达120万人。柴湖移民区、漳河库区等扶贫工作成效明显。累计解决13.1万农村贫困人口的温饱问题和7.2万人的饮水困难。就业再就业工作不断加强，城镇职工基本养老、基本医疗和失业、工伤、生育保险制度逐步健全，城镇居民最低生活保障基本实现全覆盖。人口和计划生育工作取得新进展，5个县市区整体进入全省优质服务先进县市区行列。民族、宗教、对台、外事、侨务、气象、档案、人防、地震等工作取得新成绩，妇女、儿童、老龄、残疾人等事业都有新进步。

　　精神文明和民主法制建设继续加强。公民思想道德建设扎实推进，群众性精神文明创建活动深入开展，涌现出刘芳艳等一批先进典型。深入实施《行政许可法》和《全面推进依法行政实施纲要》，自觉接受人大及其常委会的法律监督、工作监督和政协的民主监督，认真听取人大代表和政协委员的意见和建议，共办理议案3件、建议597件、提案921件。加强基层民主政治建设，乡、村换届普遍实行“两推一选”。普法依法治理工作取得成效。市场秩序进一步规范，公共安全管理得到强化。国防教育和双拥工作深入开展，国防动员和民兵预备役建设不断加强。大力推进平安荆门建设，强化社会治安综合治理，重视群众来信来访，努力保持社会稳定。加强服务型政府建设，积极推进电子政务，开通“中国.荆门”网站、“12345”市长专线和政风行风热线，建立政府新闻发言人制度，成立市行政服务中心等“五个中心”，政务、厂务、村务公开取得成效。坚持为民办实事，一些关系群众切身利益的问题得到解决。扎实开展保持共产党员先进性教育活动，廉政建设和反腐败工作稳步推进。

　　各位代表，刚刚过去的2006年，是实施“十一五”规划的起步之年。一年来，我们按照“科学发展、开放发展、创新发展、和谐发展”的要求，着力做好“打基础、管长远”的工作，结合荆门实际理清思路，制定完善规划，狠抓督办落实，努力保持工作的连续性和稳定性；着力加快结构调整，突出抓好项目建设、园区发展、招商引资、新农村建设等重点工作，促进经济平稳较快增长；着力促进社会发展和解决民生问题，扎实办好十件实事，千方百计保障困难群众的基本生活；着力保持社会稳定，加强基层基础工作，妥善处理社会矛盾，积极化解不和谐因素，较好完成了市六届人大四次会议确定的各项任务，实现了“十一五”规划的良好开局。初步核算，全年生产总值比上年增长11.3%；规模以上工业增加值增长20.3%；全社会固定资产投资127.7亿元，增长25.5%；社会消费品零售总额增长14.4%；出口总额1.29亿美元，增长34.9%，利用外资9849万美元，增长17.6%；单位生产总值综合能耗下降3.8%左右；完成一般预算收入10.17亿元，增长9%；城镇居民人均可支配收入和农民人均纯收入分别增长9.4%和8.6%；城镇登记失业率为4.1%，人口自然增长率为2.2‰。

　　回顾本届政府的工作历程，我们深切地体会到：做好新形势下的政府工作，必须坚持科学发展、共创和谐，任何时候都不能动摇；必须坚持以人为本、关注民生，任何时候都不能忘记；必须坚持深化改革、扩大开放，任何时候都不能停步；必须坚持城乡协调、统筹兼顾，任何时候都不能偏颇；必须坚持开拓创新、抢前争先，任何时候都不能懈怠。

　　各位代表，本届政府取得的上述成绩来之不易，这是省委、省政府和市委正确领导，全市人民开拓进取、团结奋斗的结果；是市人大、市政协监督支持，各民主党派、无党派人士、各人民团体，驻荆部队、武警官兵大力支持的结果。在此，我谨代表市人民政府，向全市各条战线的广大干部群众，向所有关心、支持和帮助荆门经济社会发展的社会各界人士和朋友，表示衷心的感谢，并致以崇高的敬意！

　　在肯定成绩的同时，我们也清醒地看到，政府工作还存在不少薄弱环节，经济社会发展中还存在不少矛盾和问题。主要表现在：经济结构不优，发展速度不快、质量不高，非公有制经济发展缓慢；农业产业化程度较低，农村基础设施脆弱，农民增收的长效机制尚未形成；能耗仍然过高，资源综合利用水平较低，环境保护压力增大；财源结构不尽合理，财政状况与经济发展的速度和社会发展的需求不相适应；社会不稳定因素依然存在，社会治安状况没有根本好转；政府职能转变还不到位，有的工作人员的宗旨观念、责任意识、自身素质还不能完全适应改革和发展的要求，还存在官僚主义、形式主义和腐败现象，等等。对这些问题，市政府将高度重视，进一步采取措施切实加以解决。

　　二、今后五年的奋斗目标和主要任务

　　今后五年，是荆门工业化的转型期、城市化的加速期、农业现代化的推进期、文化旅游产业化的发展期。经济全球化和区域经济一体化进程的不断加快，国际国内产业的加速转移，促进中部地区崛起、建设社会主义新农村战略决策的全面实施，为荆门未来发展提供了良好机遇。我市经过多年的建设，为加快荆门发展打下了较好基础，特别是市第六次党代会勾画了未来五年的发展蓝图，全市上下加快发展的愿望更加迫切，奋力崛起的氛围正在形成。但我们还应该看到，思想观念的差距、开放创新的差距、发展环境的差距，仍影响和制约着我市经济总量的扩张、发展质量的提高、城市形象的提升以及和谐社会的构建；周边地区你追我赶、竞相发展的逼人态势，使我们在新一轮发展中面临着更大的竞争压力。我们务必增强不进则退、小进也是退的危机感，增强责无旁贷、争先进位的责任感，在挑战中抢抓机遇，在压力下寻找出路，在困难中谋求对策，全面推进荆门经济社会又好又快地发展，为综合实力跻身全省第一方阵打下坚实基础。

　　根据市第六次党代会部署，今后五年政府工作的指导思想是：高举邓小平理论和“三个代表”重要思想伟大旗帜，全面落实科学发展观，坚持科学发展、开放发展、创新发展、和谐发展的有机统一，以加快荆门崛起为主题，以调整经济结构、转变增长方式、提高增长质量为主线，以改革开放和自主创新为动力，坚持走新型工业化道路，扎实推进社会主义新农村建设，着力促进社会发展和解决民生问题，奋力向工业强市、农业强市、旅游强市和生态宜居城市跨越，努力建设经济繁荣、城乡秀美、民生殷实、社会和谐的新荆门。

　　主要预期目标是：到2011年，全市生产总值超过600亿元，年均增长11%。规模以上工业增加值超过200亿元，年均增长16.7%。单位生产总值综合能耗比2006年下降20%，主要污染物排放总量下降10%。地域财政收入超过50亿元，年均增长14%以上。全社会固定资产投资累计超过900亿元，年均增长12%。外贸出口达到3亿美元，年均增长15%，累计吸收外商直接投资6.7亿美元，年均增长12%。社会消费品零售总额达到250亿元，年均增长12%。城镇居民人均可支配收入年均增长8%，农民人均纯收入年均增长6%。城镇登记失业率控制在4.5%以内，人口自然增长率控制在5‰以内。中心城区建成区面积超过50平方公里，人口超过50万。全市城镇化率达到46%，森林覆盖率达到38%。

　　提出上述目标，既是贯彻科学发展观和构建和谐荆门的需要，也是落实省委、省政府和市委对荆门发展要求的需要，更是实现好、维护好、发展好全市人民根本利益的需要。实现这些目标，必须采取超常规的发展措施，必须付出艰苦的努力。

　　为此，今后五年重点做好以下六个方面的工作：

　　（一）坚持走新型工业化道路，着力提高工业经济整体素质

　　优化工业结构。改造提升传统行业，扶持培育新兴行业。延伸石化产业链，以资源综合利用和精深加工为发展方向，支持中石化荆门分公司扩能改造；延伸磷化产业链，支持湖北洋丰、大峪口公司等企业实行采选、矿肥、矿化结合，整合磷化资源，努力打造全省乃至全国的重要化工基地。延伸建材产业链，支持葛洲坝水泥厂等建材企业扩张壮大，加快发展新型干法水泥、特种水泥、新型墙体材料、高档陶瓷材料等新型建材产品，努力打造湖北的重要新型建材工业基地。大力引进项目、资金、技术、智力和管理理念，着力培植纺织、食品、机械制造企业群和以新医药、新材料、电子信息为主导的高新技术企业群。进一步优化产品结构，加大企业技术改造和新产品研发力度，加强企业自主创新能力建设，大力推进产学研合作，建立企业创新基金，创立2家以上国家级、6家以上省级技术中心。引导企业实施精品名牌战略，推行国际质量、环境体系和安规认证，力争培育3个以上中国名牌产品或中国驰名商标。

　　加快园区和项目建设。打破行政区域界限，按照区域经济一体化、产业集群化、产品差异化的要求，完善园区产业规划布局，加快形成分工合理、重点突出、比较优势得到充分发挥的区域产业结构。加强园区基础设施建设，创新园区体制机制，努力把荆门经济开发区建成高新技术产业聚集区，把荆襄、宏图及各县市区工业园区建成新兴工业基地和财源基地。坚持大抓项目、抓大项目，按照抓紧“储备论证一批、跟踪落实一批、加快实施一批”的要求，进一步加大项目建设力度，做到引进一个大项目，培育一个龙头，做大一个产业，带动一方经济。

　　壮大市场主体。坚持扶优扶强，推动生产要素向优势产业、优势企业集中。支持中省企业发展改革，带动地方工业加快发展，实现互惠共赢。积极为成长型企业提供个性化服务，推进企业之间大协作、大配套，培育和发展一批“专、精、特、新”的中小企业。力争到2011年，全市规模以上工业企业超过800家，形成销售收入过200亿元、100亿元、50亿元、10亿元的企业梯队。重视和加强企业家队伍建设，建立健全培养、评价、激励、约束机制，促进企业家队伍不断发展壮大。

　　加强资源循环利用。按照“减量化、资源化、再利用”的要求，组织推广一批节能、节水、资源综合利用的新技术、新项目。以湖北洋丰、格林美、葛洲坝水泥厂、泰山建材等企业为重点，推进产业循环式组合，努力形成5-10个产业循环式组合链条。抓好石油、化工、建材等行业以及年耗万吨标煤以上企业的节能降耗工作，加快淘汰高耗能、高耗水、高耗材、高污染的工艺、设备和产品。推进工业废弃物综合利用，加强生活垃圾资源化利用和污水处理设施建设。

　　（二）大力发展现代农业，扎实推进新农村建设

　　调整农村经济结构。大力发展板块经济，抓住市场化取向、区域化布局、专业化种养、标准化生产四个关键环节，促进优势特色农产品向优势产区集中。围绕年产100万吨优质米、100万锭棉纱、30万吨名特优水产品、50万吨优质油、500万头猪、5000万只家禽、15万吨燃料乙醇、20万立方米优质中高密度纤维板的发展目标，稳定粮食生产，扩大优质稻、优质棉、“双低”油菜种植面积，加快发展畜牧业和名优水产业，加强速生丰产林基地建设。大力发展无公害农产品、有机食品、绿色食品，提高农产品质量。大力发展特色经济，扩大蔬菜、花卉苗木、水果种植面积，发展特种养殖业，鼓励农民开展小加工、小经营，形成“小产品、大产业”、“小商品、大市场”的发展格局。大力发展劳务经济，加强农村劳动力技能培训，积极培育劳务品牌，引导农村劳动力向非农产业和小城镇转移，引导有资金、有技术的外出务工人员回乡创业，五年转移农村劳动力新增20万人以上。

　　壮大农业产业化龙头企业。坚持以工业化带动产业化，依托优势产业和特色农产品发展壮大龙头企业，努力把荆门建设成为湖北重要的优质农副产品加工和出口基地。鼓励龙头企业打破所有制、行业、地域界限，通过兼并、收购、重组等形式扩张发展。围绕实施“一袋米”等工程，扶持国宝桥米等公司加快发展，整合粮食加工企业，争创中国名牌产品；扶持中粮祥瑞、金龙泉、宝源集团、众和纺织、华溢纺织等企业延伸产业链，开发高附加值产品。积极引进畜产品、水产品加工骨干企业，支持神地科贸、仙羽禽业等企业加快发展。力争到2011年，建成3-5个国家级农业产业化龙头企业，全市农副产品加工业产值增加100亿元以上。

　　改善农业生产和农民生活条件。科学制定和实施乡村建设规划，加强乡村道路、安全饮水、电网改造、小水电和沼气等工程建设。到2011年，全市所有乡镇通三级路，村村通油路(水泥路)，有条件的硬化到自然村；全面完成农村安全饮水工程；新增户用沼气池10万口以上。继续加强农村水利基础设施建设，着力推进“三民水利”。加强农业综合开发，提高防灾减灾能力。严格保护基本农田，加强土地整理和复垦工作，确保耕地总量动态平衡。积极探索建立“依法、自愿、有偿”的土地流转机制，促进土地适度规模经营。

　　积极发展农村公共服务事业。促进公共财政向农村倾斜，公共服务向农村覆盖。进一步完善“以钱养事”新机制，健全农村公益性服务体系，大力扶持创办各类经营性服务组织，在防病治虫、测土施肥、供种供苗、农机服务、科技信息等方面为农民提供全程服务。加强农村市场建设，完善农村流通网络。积极鼓励供销、粮食、科协等部门参与农村合作组织建设，大力发展农产品购销大户、经纪人队伍和流通服务组织。加快农机化发展，力争到2011年，农机化综合水平达到68％。建设农产品质量安全监督检测体系，加强动植物疫情监测，强化产地检疫。认真落实各项惠农政策，加强农民负担监管，严禁新增并稳妥化解村级债务。

　　（三）提升服务业发展水平，发展壮大第三产业

　　突破性发展文化旅游业。加强对旅游资源的科学规划和合理开发，构筑政府主导、部门配合、社会参与、市场运作的大旅游格局。大力实施“143”旅游发展战略，按照“整合资源、培育精品、打造品牌、形成网络”的发展方向，着力构建明显陵和黄仙洞、汤堰温泉和绿林景区、漳河和荆门城区三大旅游板块，推动旅游业向观光度假、康体休闲为一体的复合型旅游转变。加快荆门旅游大环线建设，开发旅游精品线路，培育荆门特色旅游品牌。改善旅游人文环境，完善旅游配套设施，加快旅游商品开发，依托职业教育资源培训旅游人才，提高旅游服务能力。加大市场营销力度，加强与武汉、宜昌等旅游市场区域合作。深度挖掘“荆楚文化”、“三国文化”、“象山文化”、“屈家岭文化”等历史文化内涵，积极促进旅游与文化、体育等产业的融合。培育发展健身娱乐、竞技表演、技术培训等体育产业，使荆门逐步发展成为“适宜于运动健身、适宜于常有赛事”的城市之一。

　　加快发展现代服务业。加强金融生态环境建设，鼓励金融机构加大有效信贷投入，积极引进各类金融机构到荆门拓展业务。建立多种形式的融资平台和信用担保体系。努力发展信息服务业，加快宽带通信网、数字电视网和下一代互联网的融合，推进“数字荆门”建设。建设现代化物流基础设施，扩大运输服务能力和范围，引进培育3-5家大型综合性现代物流企业，建设3-4个特色物流园区。培育发展各类中介服务业，鼓励公平竞争。

　　改造和提升传统服务业。加强城区商业网点、专业市场、特色街及其他商业设施的规划、建设和管理，做大做强商品批发市场，把荆门打造成为区域性商品批发中心。支持大型商业集团向农村延伸，发展乡级连锁超市和村级便民店1000家以上。大力发展社区服务业。促进房地产业健康发展，重点发展面向中低收入家庭的商品住房、经济适用住房，建立健全廉租住房制度，积极发展健康环保和节能省地型住宅，规范发展物业管理。

　　（四）加快城市化进程，建设生态宜居城市

　　加快中心城区建设。按照“依托老城、建设新城、新老一体、联动发展”的原则，高起点规划、高品位建设新城区，高标准配套供水、供电、供气、通信等服务设施，做到“开发一片，建成一片，绿化、美化、亮化一片”，塑造现代化城市新形象。实行统一规划，分区发展，责利挂钩，合力建城，调动市、区两级和市直有关部门的积极性，共同推动新一轮城市建设。进一步完善城市功能，提升城市品位，努力形成历史文化、现代文明交相辉映，新老城区优势互补、各展风采的城市风貌。强化规划的龙头作用，严格规划管理，维护规划的权威性和严肃性。改革城市管理体制，落实长效管理机制，创新城市管理手段，运用数字化等先进技术提升城市管理水平。充分发挥区、街道和社区在城市管理中的作用，进一步改善城市环境面貌，争创国家园林城市、卫生城市。

　　优化城镇布局。加快钟祥、京山、沙洋城区建设，增强综合服务功能，强化集聚和带动作用。积极发展胡集、后港、宋河等一批重点小城镇，着力发展李市、张集、曹武、马河等口子镇和绿林、漳河、客店、纪山、易家岭等旅游镇，建设一批各具特色的小城镇。加快城区与周边乡镇基础设施的延伸配套。优化村庄布局，加快中心村建设。

　　加强生态环境保护。严格实施环境容量总量控制、环境影响评价和“三同时”制度，坚决控制环境污染，争创环保模范城市。禁止新上污染项目，关停并转高污染企业。加强饮用水源保护，强化漳河、汉江饮用水取水点、惠亭水库、温峡水库、长湖等水源地保护。加大环境监察力度，认真抓好竹皮河、京山河、西荆河、钟祥护城河等水污染防治工作，对汉江流域荆门段各支流水系水质实行分段达标管理。加大重点污染源治理力度，实现主要污染物逐年减排。加强城市污水处理，污水处理率达到70%。合理施用农药、化肥，切实防治农村面源污染。积极实施天然林保护、长江防护林、退耕还林还草、湿地保护等重点生态工程。加强东宝山、象山、圣境山、罗汉山等城市森林保护，坚决制止乱砍滥伐，严禁违规开山采石。整顿和规范矿产资源开发秩序，促进资源的科学保护与节约利用。

　　加强基础设施建设。积极服务荆门境内高速公路建设，力争2010年前全部通车。加强以中心城区为轴心的交通环网建设和县乡公路建设，实现县县通高速公路或一级公路。进一步改善城乡电网设施，服务荆门电厂“大代小”四期、荆门1000千伏特高压等电力工程建设。推进天然气工业利用，改善能源结构。加强城市消防和人防设施建设。

　　（五）推进体制机制创新，增强发展活力

　　继续深化各项改革。深化行政管理体制改革，推进政府管理创新。深化企业改革，以建立现代企业制度为目标，完善公司法人治理结构。加大国有资产优化配置力度，进一步完善国资监管体制。推进城市公用事业改革，探索公共服务市场化运作方式。深化农村综合改革，切实抓好县乡财政体制、农业“小三场”、集体林权制度等改革。加快建设公共财政体系，完善部门预算、国库集中收付、政府采购和收支两条线管理制度，逐步提高各类公共服务的支出比重。深化投融资体制改革，规范政府投融资行为，确立企业投资主体地位。整体推进事业单位分类改革。深化文化体制改革，构建公共文化服务体系。深化要素市场改革，积极培育劳动力、技术、土地、产权、信息等要素市场。

　　提高对外开放水平。加大招商引资力度，落实激励政策，积极推行专业招商、产业招商、企业招商等招商方式，抓好项目跟踪落实服务工作。积极应对国际贸易环境变化，转变外贸增长方式，优化出口商品结构，加强机电产品、农副产品、轻工产品、轻纺产品和医药产品等出口基地、出口品牌建设，培育一批年出口额过千万美元的出口企业。加强区域经济合作，实施“走出去”战略，支持和引导优势企业到市外、境外开展加工贸易、资源开发和对外工程承包与劳务合作。主动融入武汉城市圈发展，加强与协作区城市互惠合作，做好产业、市场和基础设施对接，推动资源联合开发。加快海关建设，设立商检机构。全方位对外宣传荆门，提高城市知名度。

　　实施全民创业工程。建立健全政府鼓励、社会支持、市场调节的创业机制，营造尊重人才、尊重劳动、尊重创造、褒奖成功、宽容失败的和谐创业氛围。激活创业主体，鼓励科技人员、高校毕业生、外出务工人员、下岗职工、离退休人员以多种形式领办、创办经济实体。加大对创业的支持和投入力度，采取财政贴息、国资参股引导、信用担保等办法，支持企业加快发展。拓展创业空间，盘活民间资本，支持民间资本“非禁即入”。坚持“以实力排座次、以贡献论英雄”，加强和改善对非公有制经济的服务，促进非公有制经济快速发展。

　　切实改善投资环境。着力建设优化环境的长效机制，综合运用行政、法律、经济和舆论等手段，以行政管理、执法部门和窗口行业为重点，以简政放权为突破口，完善制度，优化服务，努力营造宽松的政策环境、优质的服务环境和诚信的人文环境，促进各种生产要素涌入集聚、各类市场主体竞相发展。

　　（六）坚持以人为本，建设和谐荆门

　　健全就业和社会保障体系。到2011年，城镇新增就业14万人，基本缓解就业难问题。不断扩大社会保险覆盖面，全市基本养老、基本医疗和失业、工伤、生育保险参保人数分别达到25万人、41万人、18万人、20万人和15.5万人。加快城乡特殊困难群众社会救助体系建设，完善城乡低保、农村五保户供养、特困户救助、灾民救济等制度。全面实施城乡困难群众医疗救助。充分发挥商业保险的补充作用。认真落实优抚安置政策，加强社会福利机构建设，积极探索养老服务新模式，发展慈善事业。继续加大对柴湖移民区、漳河库区、贫困山区、革命老区的扶贫力度。健全住房公积金制度，切实改善中低收入群众的居住条件。

　　大力发展各项社会事业。坚持实施科教兴市和人才强市战略，加大科技投入，完善技术创新成果作为生产要素参与收益分配的机制，建立科技创新、成果转化、资源共享、创业融资等平台，建设创新型荆门。继续实施“113”人才发展计划，加快培养引进高层次、高技能、复合型人才，加强农村教师、卫生医疗和农业科技队伍建设，建立健全人才选拔、使用和激励的新机制。巩固义务教育成果，基本普及高中教育，大力发展职业教育，推进素质教育和创新教育，支持荆门本科院校建设。加强公共卫生服务体系建设，完善城市社区及县乡村三级医疗服务网络，全面实施新型农村合作医疗制度。加快突发公共卫生事件应急体系和卫生监督体系建设，做好重大传染病的预防和控制。进一步繁荣文化事业，大力发展文学艺术、新闻出版和广播电视等文化产业。深入开展“扫黄打非”，增加基层科学文化活动网点，培育和发展健康的文化市场。加强文物保护和非物质文化遗产保护，改造扩建市博物馆、图书馆等文化设施。加强乡镇、社区综合文化站和村组文化活动室建设，活跃群众文化生活。大力发展群众性体育活动，提高竞技运动水平，承办好湖北省第十三届运动会。发展广播电视事业，推进广播电视数字化，努力实现广播电视“户户通”。认真做好人口和计划生育工作，稳定低生育水平，改善出生人口素质和结构。维护和保障妇女、儿童、老年人合法权益，积极发展残疾人事业。做好社会科学、方志、档案、保密、民族、宗教、对台、外事、侨务、气象、地震等工作。

　　加强精神文明和民主法制建设。深入推进社会主义荣辱观教育，大力倡导社会主义核心价值观，广泛开展群众性精神文明创建活动，全面提升市民素质和城乡文明程度。重视支持关心下一代工作，关爱农村留守学生成长，抓好未成年人思想道德建设，预防青少年犯罪。加强基层民主政治建设，完善村民自治、社区自治，大力推行政务、厂务、村（居）务公开。改善社区工作条件，完善社区服务和活动设施。全面推进“和谐村镇”建设。积极发挥社团组织在构建和谐社会中的作用。加快依法治市进程，深入开展“五五”普法教育。贯彻落实监督法，自觉接受人大及其常委会的监督，支持人民政协履行政治协商、民主监督和参政议政职能，认真办理人大议案、代表建议和政协提案，切实提高办理落实率。认真听取各民主党派、无党派人士和人民团体意见，坚持和完善政府与民主党派的联系制度。维护司法公正，建立以政府投入为主的司法救助和法律援助经费保障机制，加大对困难群体特别是农民工法律援助力度。

　　努力维护社会稳定。高度重视人民群众来信来访，建立健全畅通的民意表达机制、有序的利益诉求机制，引导群众以理性合法的方式表达利益诉求。深入开展“百千万”矛盾纠纷排查调处活动，通过调解、仲裁、行政复议等多种方式，预防和依法处理各种社会矛盾，妥善处理群体性事件。加强社会治安综合治理，依法严厉打击严重刑事犯罪活动。健全统一指挥、反应灵敏、协调有序、运转高效的应急反应机制，提高政府应对公共危机的能力。加强价格监测与管理，整顿和规范市场秩序。坚持“安全第一、预防为主、综合治理”方针，加大安全生产监管和执法力度，坚决防止和减少各类重特大事故发生。加强国防动员和人民防空、民兵预备役工作，巩固双拥创建成果，促进国防建设与经济建设协调发展。

　　三、2007年的重点工作

　　2007年是落实科学发展观、奋力加快荆门崛起的第一年，也是市七届人民政府工作的起步之年。起步关乎崛起，开局影响全局。能否抢抓机遇、加快发展，是对我们行政能力的严峻考验。我们必须以时不我待、只争朝夕的精神，以勇于突破、抢前争先的决心，以锐意进取、勤奋扎实的工作，坚持抓基层、打基础；抓重点、破难点；办实事、求实效，努力实现良好开局。

　　今年全市经济社会发展的主要预期目标是：生产总值增长11%；单位生产总值综合能耗下降4%，主要污染物排放总量下降2％；规模以上工业增加值增长18%；全社会固定资产投资增长18%；社会消费品零售总额增长13%；吸收外商直接投资增长15%，外贸出口增长15%；地方一般预算收入增长12%；城镇居民人均可支配收入增长8%，农民人均纯收入增长6%；城镇新增就业3万人，登记失业率控制在4.4%以内；人口自然增长率控制在3.5‰以内。

　　（一）以提高质量效益为中心，加快工业经济发展。一是大力扶持重点骨干企业。采取领导挂点联系、部门结对帮扶、银行对口支持、优惠政策倾斜、资源优先配置等办法，在全市重点调度47家企业，促其达产达效。以资本为纽带、产品为依托，引导资本、技术和人才向优势企业聚集，尽快形成一批主业突出、核心竞争力强的“旗舰型”企业集团。二是大力实施企业成长工程。设立科技型中小企业创新基金，采取技改贴息、国有资本介入引导等办法，积极支持市场前景好、产品附加值高、税收贡献大、污染小、能耗低的中小企业加快发展，着力壮大格林美、维益电子、新美香、新宇机电等成长型企业，力争全市新增规模以上工业企业100家以上。三是大力发展企业集群。围绕建设“两大基地”、“四大企业群”目标，破除区域界限，探索项目异地落户的税收分成、产值分计等办法，鼓励企业按产业规划到园区落户。综合运用市场、经济、行政、法律手段，大力治理“散、乱、低”现象，发展企业集群、产业集群，力争年内荆门经济开发区和各县市工业园区新增规模以上企业均超过10家。进一步整顿规范磷矿等矿产资源开发秩序，加快推进矿山企业改革重组。四是大力实施项目带动。按照“一个产业、一批项目、一名领导、一个专班、一笔经费”的要求，加大招商引资力度，创新招商方式，引进一批企业落户荆门。积极做好荆门电厂三期扩建、中石化荆门分公司扩能改造工程等续建项目的服务工作，确保荆门电厂三期第二台机组按期投产发电；促进天茂集团二甲醚、中磷公司采选矿工程、东光公司整体迁建等项目年内竣工；启动15万吨燃料乙醇、葛洲坝水泥厂2条日产5000吨水泥熟料生产线等重大项目；做好荆门电厂“大代小”四期、中石化荆门分公司四氧化炭项目的前期工作。五是大力培育精品名牌。重点支持“国宝”桥米、“洋丰”磷复肥、“宝源”中高密度纤维板等争创中国名牌产品或中国驰名商标。加快培育出口主体和出口品牌，扶持京山轻机、中粮祥瑞、普安阀门、天茂集团、金龙非织造布等企业扩大出口规模，积极支持六○五所、湖北洋丰、维益电子、宏图等企业开展自营出口业务。六是大力推进节能降耗。科学编制循环经济发展总体规划，支持试点单位建设循环经济项目。推进化工、建材、电力等传统行业开展清洁生产，重点抓好20家耗能大户的监督管理，有效降低能耗、物耗，依法依规淘汰落后生产能力。同时，积极做好煤电油运和原材料等要素资源的协调与科学调配，保障企业正常生产经营。认真抓好亏损企业扭亏增盈工作。

　　（二）以增加农民收入为核心，加快发展现代农业。一是着力壮大板块基地。坚持统筹规划、科学布局、连片种植，抓好200万亩优质稻种植，扩大有机稻种植面积10万亩；抓好50万亩优质棉花种植，其中彩棉2000亩；抓好180万亩“双低”油菜、6000亩红薯生产示范基地和200万亩速生丰产林基地建设。新建畜禽养殖小区20个，生猪出栏达到310万头，家禽出笼达到3300万只。抓好五大水产片带建设。二是着力壮大龙头企业。落实财政专项资金和税收减免政策，采取联合重组、股份兼并、品牌授权等方式，积极推进企业融合、资源整合和产业优化，依托一个产业培植一家大型龙头企业。强力实施“一袋米”工程，力争国宝桥米年产量达到50万吨，年产值超过15亿元；推动中粮祥瑞壮大规模、扩大出口；支持宝源集团延长产业链；促进众和纺织做大做强、华溢纺织由纺纱向织布发展。积极探索龙头企业与农民风险共担、利益均沾的联结机制，实现企业与农民的双赢。大力发展农产品购销大户、经纪人队伍和流通服务组织，重点抓好100个农民专业合作经济组织。三是着力加强农村基础设施建设。抓好石门、陈坡等5座大中型水库除险加固，改造104座泵站，改造硬化末级渠系200公里，建设高产农田10万亩以上，整理、复垦土地1万亩。新建沼气池2万口。筹资7800万元实施“农村安全饮水”工程，解决20万人的饮水安全问题。以列入全省“百镇千村”建设的4个镇、59个村和全市“十村示范、百村整治”的村为重点，开展“城乡清洁工程”。推进农村低压电网改造工程建设，启动20户以上自然村广播电视“村村通”工程。四是着力加强农村公益性服务。将农村公益事业服务经费纳入年度财政预算，按照种植业每亩不低于1元、畜牧防疫每户不低于2元、文化体育每人不低于0.5元的标准，加大对农村公益性服务的投入，提高农村公益性事业服务质量和效率。扶持发展乡镇综合文化站、村级科技文化活动中心和科技文化中心户，不断丰富农民业余文化生活。五是着力加强劳动力培训转移。整合农业、教育、科技、劳动保障等部门培训资源，把培训与促进创业、推进农业产业化、发展劳务经济有机结合，力争全年培训农民10万人次以上，转移农村劳动力新增4万人，人均劳务收入增加200元以上。

　　（三）以提升城市功能和美化环境为重点，加快城乡建设步伐。一是拓展城市发展空间。全面启动新城区建设，增强城市产业集聚能力，重点抓好城区主干道路、公共活动场所、公用停车场、标志性建筑物建设。以承办“八艺节”分会场为契机，加强体育文化设施建设，美化城市面貌。着力提升老城区市政设施档次，增加市区绿地面积。加强东宝山公园、虎牙关水上乐园建设，改造并免费开放龙泉公园。扩建市科技馆。继续加强城区出口道路改造。以强化产业支撑、完善城镇功能、改善人居环境、提高管理水平为重点，加快构建布局合理、功能互补的城镇体系，推进产业、人口、发展要素向各县(市)城区和小城镇聚集，增强区域带动力。二是加快服务业发展。完善旅游规划，整合旅游资源，引进1-2个战略投资者开发城区文化旅游项目。打造1-2个4A级景区，建设2-3家四星级以上宾馆，鼓励景区发展家庭旅馆。积极推进大洪山环线旅游项目建设，改造旅游公路。深度开发旅游产品，进一步开发休闲度假、生态旅游、红色旅游和观光农业，发展“农家乐”等乡村旅游。积极发展商贸流通业，完善城区商贸网点规划，完成1家城区大型集贸市场建设，增加零售市场网点，建设1家区域批发市场。加快“万村千乡”市场工程进程，新建农家店240家。积极促进体育健身、文化休闲等服务业发展。三是加强城乡环境综合整治。深入开展“城市管理年”活动，继续整治市容、交通、经营、治安等秩序。提高城建规划水平，强化规划监管，严肃查处各类违法违规建设行为。制定饮用水水源地保护规划，进一步加强饮用水源保护。加大重点污染源治理力度，对工业污染大户实施在线监控，建立环保有奖举报制度。启动新城区和京山污水处理厂建设，建成市医疗废物处置中心和钟祥污水处理厂。综合整治城市环境，切实解决群众反映强烈的饮食业油烟、社会生活噪声、建筑施工扬尘以及城区周边开山采石、劈山建房等突出问题。加强乡村清洁示范村建设，实施农村小康环保行动计划。

　　（四）以构建公共财政为目标，加快培植和壮大财源。把发展工业经济、扩大招商引资、推进全民创业放在突出位置，努力培育一批新的税源增长点，逐步建立主体税源坚实、地方税源充足的财源体系，努力提高财政收入占生产总值和工商税收占财政收入的比重，确保财税收入增幅高于经济增长速度。综合运用预算、税收、贴息、担保等手段，支持产业结构调整和高新技术产业发展。坚持依法治税，强化税收征管，正确处理依法征收与优化经济环境、培植税源的关系，严厉打击骗税、偷税、逃税等违法行为，确保应收尽收。加强对矿产资源开采、商贸流通、土地使用、交通运输、房地产交易以及零星税种的征管。坚持公共财政方向，优化支出结构，扩大社会保障支出，加大对新农村建设和社会事业的财政投入，重点保证农业和农村基础设施、农村基础教育、城乡公共卫生、基层文化事业支出需要。进一步规范部门预算和国库集中收付，完善政府采购制度，认真落实收支两条线管理制度。牢固树立过紧日子的思想，严格规范和控制公务接待费支出，严禁滥发津补贴，严格编制管理。加强财政专项资金监督，增强财政资金的规范性、安全性、有效性和透明性。扎实做好金融工作。进一步密切银政、银企关系，促进金融与地方经济互动双赢。积极推动国有商业银行、农业发展银行和农村信用社健康发展，鼓励保险、证券等金融机构拓展业务。加强中小企业信用担保工作，采取多种形式扩大担保公司规模。认真做好不良资产打包处置工作。继续推进信用荆门建设，引导信贷资金投向高新技术产业、农业产业化龙头企业、文化旅游业和城乡基础设施建设。

　　（五）以实施“六大民心工程”为突破口，加快建设和谐荆门。一是实施扩大就业工程。落实再就业政策，积极开发公益性岗位，帮助下岗失业人员再就业1.5万人、困难人员再就业3000人，年内基本解决有就业愿望的“零就业家庭”就业问题。二是实施社会保障工程。各项社会保险扩面新增3.2万人次。将城区青少年学生和达到法定退休年龄的城区居民纳入医疗保险。全面启动农村最低生活保障制度，按每人每月不低于20元的标准实行分类救助。完善城乡贫困群众医疗救助制度，筹措500万元，对城镇低保对象、五保户、特困户、特困优抚对象中患重大疾病的人员实施医疗救助，资助5万名农村五保户、特困户参加新型农村合作医疗。做好大中型水库移民后期扶贫工作，启动南水北调中线工程移民安置工作。三是实施公平教育工程。落实农村义务教育经费保障机制，按每生每年不低于15元的标准给予公用经费补助。支持80所农村中小学实施改水项目、60所学校实施改厕项目。巩固提高“普九”成果，迎接国家验收。坚决制止义务教育阶段学校乱办班行为。强化学校安全教育和管理。春节前集中时间普遍走访农村留守学生。发展职业教育，筹措500万元支持市本级中等专业学校建设，资助2500名家庭困难学生接受中等职业教育。加强农村师资队伍建设，接收20名以上高校优秀应届毕业生、选派100名市县城区优秀教师到农村定期支教。增加荆门本科院校建设投入。四是实施全民健康工程。在荆门城区建好4个社区卫生服务中心和38个社区卫生服务站，开展社区卫生服务“首诊制”试点，社区卫生服务覆盖面达85%。在城区2家惠民医院规范开展惠民医疗服务，在县市区综合医院开设惠民病房或惠民门诊。努力扩大农村养老保险和新型农村合作医疗覆盖范围。争取国债资金500万元，完成10所乡（镇）卫生院的改扩建任务，优化村卫生室配置，村卫生室合格率达70%。开展晚血病人救治、人群化疗和药物灭螺，晚血病人免费救治率达100%。春节前后对返乡农民工普遍进行一次体检。五是实施交通畅通工程。加快农村公路发展，投资1.69亿元改造县乡公路88公里、建设通村四级以上油路（水泥路）500公里。六是实施市民安居工程。完善住房保障制度和住房公积金制度，中心城区新建经济适用住房2万平方米，重点解决人均住房面积不足10平方米家庭的住房困难。在实施“六大民心工程”的同时，做好维护稳定和安全生产工作。加强市县乡三级群众来访接待中心建设，畅通群众诉求渠道。对外出务工人员普遍进行走访慰问，了解其需求，积极维护其合法权益。扎实抓好平安创建活动，加强社会治安综合治理，依法严厉打击“两抢一盗”、黑恶势力、吸毒贩毒、地下“六合彩”等违法犯罪活动，下大力整治企业、中小学校周边环境，增强人民群众安全感。加大安全生产监管和执法力度，切实抓好接送学生车辆、矿山、危化品、建筑施工以及公众聚集场所消防安全等专项安全整治，强化食品药品监管，确保人民生命财产安全。

　　四、切实加强政府自身建设

　　站在新的起点，政府肩负着发展的重任，承载着群众的厚望。我们必须进一步强化创新意识、服务意识、责任意识，加快政府职能转变，推进行政管理创新，努力建设一个干事创业、奋发有为、务实创新、廉洁高效和人民满意的政府。

　　----解放思想、开拓创新，塑造作为政府的新形象。牢牢把握发展主题，坚决破除影响发展的思想观念、思维方式、体制障碍，坚决改变束缚发展的规定和做法，主动把荆门的发展放在全国、全省的大局中来谋划，向改革要办法，因地制宜找准加快发展的切入点；靠创新找出路，打开推动发展的突破口；以实干求实效，落实促进发展的新举措。积极开展“机关比服务、企业比贡献、乡村比发展、群众比致富”活动，建立目标责任考核制度，强化约束激励机制，把全市上下的注意力引导到干事创业上来，把社会各界的积极性凝聚到加快荆门崛起上来。

　　----以人为本、勤政为民，塑造服务政府的新形象。进一步强化“民本”意识、“公仆”观念，自觉把维护好、实现好、发展好最广大人民群众的根本利益作为制定政策、开展工作的出发点和落脚点，一切依靠人民，一切为了人民，逐步提高人民群众的满意度和幸福指数。切实转变政府职能，把工作重点放在规划指导、政策引导、社会管理、公共服务和创造优良的经济发展环境上。继续深化行政审批制度改革，规范行政许可行为，完善行政服务中心功能，加快推行网上审批、网上办事、互动交流，最大限度地减少办事环节、缩短办事时限。加大政务公开力度，进一步健全政府重大事项专家咨询、社会公示、听证制度以及社情民意反映制度，提高政府科学民主决策水平，增强政府执行力和公信力。

　　----转变作风、真抓实干，塑造务实政府的新形象。咬定发展目标不动摇，抓好各项决策和工作部署的检查落实，确保政令畅通。加强行政效能建设，精简会议、文件，减少应酬、接待，改进会风、文风，防止急功近利，力戒心浮气躁，克服官僚主义和形式主义，多做“打基础、管长远”的工作，把更多的时间和精力用于深入基层、调查研究、解决问题上。敢于承担责任，敢于较真碰硬，努力发挥政府部门的整体功能，形成推进工作的强大合力。

　　----依法行政、规范管理，塑造法治政府的新形象。把依法行政作为政府工作的基本准则，严格依照法定权限和程序行使权力、履行职责、接受监督。加大行政综合执法改革力度，加快推进相对集中行政处罚权的改革工作，完善行政复议、行政赔偿等制度。严格落实行政执法责任制，强化行政执法监督，坚决克服多头执法、执法不公、执法违法现象，严肃查处不作为、乱作为、慢作为行为。

　　----廉洁从政、从严治政，塑造廉洁政府的新形象。坚持标本兼治、综合治理、惩防并举、注重预防的方针，深化源头治腐，加强廉政建设和反腐败斗争。严格执行领导干部廉洁从政的各项规定，加强对权力运行的制约和监督。加强行政监察、审计监督和政府督查，坚决查处违纪违法案件，坚决惩处腐败分子，坚决纠正损害人民群众利益的不正之风。厉行节约，反对铺张浪费，以节约型政府带动节约型社会建设。加强公务员队伍的教育、管理和监督，努力提高公共管理服务的能力和水平，建设高素质的公务员队伍。

　　各位代表，加快荆门崛起需要我们共同开拓，构建和谐荆门需要我们加倍努力。我们坚信，通过全市人民的团结奋斗，本次大会确定的各项目标一定能够实现。让我们紧密团结在以胡锦涛同志为总书记的党中央周围，高举邓小平理论和“三个代表”重要思想伟大旗帜，在省委、省政府和中共荆门市委的正确领导下，紧紧依靠全市人民，充分调动一切积极因素和发展力量，抢抓机遇，励精图治，为加快荆门崛起而努力奋斗！
